# Efficacy and safety of phenobarbital for benign convulsions with mild gastroenteritis: A prospective randomized controlled study

**DOI:** 10.1097/MD.0000000000031495

**Published:** 2022-12-16

**Authors:** Jian Zha, Yong Chen, Xiongying Yu, Jihua Xie, Zhaoshi Yi, Hui Chen, Jianmin Zhong

**Affiliations:** a Nanchang University, Nanchang, China; b Department of Neurology, Jiangxi Provincial Children’s Hospital, Nanchang, China.

**Keywords:** convulsions, mild gastroenteritis, phenobarbital (PB), randomized control trial, safety, efficacy

## Abstract

previous studies have shown that phenobarbital (PB) is a effective and safe drug in the treatment of benign convulsions with mild gastroenteritis (CwG), but there is a lack of large sample prospective randomized controlled study of different doses. This study was a prospective randomized controlled study on the efficacy and safety of different doses of phenobarbital for CwG. There has been no similar study.

**Methods:**

One hundred twenty CwG cases were included in this study. All of them were hospitalized in the Department of Neurology of Jiangxi Provincial Children's Hospital from January 2019 to August 2021. They were randomly divided into 10 mg/kg single dose group (Group A, n = 60) and 5 mg/kg single dose group (Group B, n = 60). The criteria for judging the efficacy of PB in our study were there was no convulsion in the course of acute gastroenteritis within 2 weeks after using PB.

**Results:**

The effective rate was 93.33% in group A and 80.00% in group B. There was significant difference between the 2 groups (*P* < .05). Drowsiness was the most frequent adverse reaction. 14 cases in group A and 7 cases in group B had drowsiness. There was no significant difference between the 2 groups in the incidence of adverse events such as somnolence, ataxia, abnormal liver function, anemia, abnormal leukocyte, respiratory depression, cognitive impairment, rash, abnormal platelet and abnormal renal function (*P* > .05). All side reaction were transient.

**Conclusion:**

it is suggested that PB 10 mg/kg intravenously should be used as soon as possible for CwG, which has high effectiveness and safety.

## 1. Introduction

Benign convulsions with mild gastroenteritis (CwG) is a common cause of convulsion caused by acute gastroenteritis, which was first proposed by Japanese scholar Morooka in 1982.^[1]^ Most of them occur in winter or spring season, in infants, showing mild gastroenteritis symptoms (such as diarrhea and/or vomiting), followed by afebrile convulsion. Most children have multiple convulsions without hypoglycemia and electrolyte disorder. The long-term prognosis of CwG is good and which does not affect growth and development. At present, it is generally considered that long-term anticonvulsant treatment is not necessary.^[2–6]^

However, due to repeated seizures in the acute phase of the disease, it brings great panic to the parents. It is recognized that acute phase treatment is needed.^[4–9]^ At present, there is no consensus on the application of CwG anticonvulsant drugs in acute phase (drug type selection, course of treatment and dose). Some scholar found that lidocaine, carbamazepine and fosphenytoin have better curative effects.^[10–12]^ In 2012, Takami et al Found the effective rate reached 100% that 24 patients with CwG were given 10 mg/kg phenobarbital (PB) intravenously at one time, and 5 cases had drowsiness and 2 cases had ataxia. Although there were adverse reactions, most of them were temporary and reversible.^[13]^ Subsequently, in 2019, that scholar conducted a randomized controlled study for the first time. The results showed that there was no recurrence of convulsions in the PB group, while 83.0% (5/6 cases) in the placebo group had convulsions again, and there was no further convulsion in these children who were given 10 mg/kg PB. There was no significant difference in the adverse reactions between the 2 groups.^[14]^ These 2 studies suggest that intravenous 10mg/ kg PB has good efficacy and safety for CwG. However, there is no comparative large sample study on the effectiveness and safety of different doses of PB.

In our previous retrospective study, we summarized 139 cases treated in our hospital. It was observed that the effective rate of 10 mg/kg single intravenous injection was equivalent to that of 5 mg/kg. recommend 5 mg/kg single administration to prevent convulsion.^[7]^ However, there were research defects in previous studies. For example, there was a large difference in the proportion between the 2 groups. There were only 40 cases in the 10 mg/kg group, and the effective rate were 92.5%, while 56 cases in the 5 mg/kg group, and the effective rate was 82.1%. Although there was no statistical difference, the effective rate of the 10 mg/kg group is higher than the 5 mg/kg group. In the later clinical work, it was found that many convulsions still occurred again after a single administration of 5 mg/kg, which greatly increased the panic of parents. Therefore, 120 cases were included in this study through a prospective randomized controlled study. They were randomly divided into 10 mg/kg single dose group (A group) and 5 mg/kg single dose group (B group). The efficacy and safety of different doses of PB were evaluated again. This study has a large number of samples and is the first prospective randomized controlled study of different doses of PB in the world.

## 2. Research object and method

### 2.1. Research object

All cases were hospitalized in the Department of Neurology of Jiangxi Provincial Children's Hospital from January 2019 to August 2021. The study has been approved by the medical ethics committee of the hospital and the informed consent of the child’s guardian.

CwG inclusion criteria^[3,14]^: vomiting, diarrhea and afebrile convulsion in previously healthy infants aged 6 months to 3 years; No dehydration or only mild dehydration, but no acidosis and electrolyte disorder; Convulsion occurs in the process of gastroenteritis. Convulsion can occur single or multiple times in one course of disease; The interictal electroencephalogram was normal or only transient background abnormality, and there was no epileptiform discharge; Serum electrolyte, blood glucose were normal.

Exclusion criteria: children with previous neurological and mental diseases; the cases who had used anticonvulsant drugs before admission; Medical history was incomplete; The child’s guardian agreed and was willing to follow up.

### 2.2. Method

#### 2.2.1. *grouping and administration method*.

There were 122 patients who met the inclusion criteria; however, medical history was incomplete in 2 of them. Ultimately, 120 cases that finally met the above criteria were randomly divided into 2 groups: A group: give PB 10 mg/kg single intravenous injection, immediately upon admission; B group was given PB 5 mg/kg single intravenous injection immediately upon admission; If the convulsion occurs again, all patients shall be given 5 mg/kg PB once again.

#### 2.2.2. *Treatment effectiveness evaluation*.

The criteria for judging the efficacy of PB were improved with reference to the previous standards.^[11,13]^ Effective: after using PB, there was no convulsion in the course of acute gastroenteritis within 2 weeks; Ineffective: there were still convulsions in the course of acute gastroenteritis.

#### 2.2.3. *Treatment safety assessment*.

Adverse reactions were observed, including drowsiness, ataxia, respiratory depression, cognitive impairment, rash, anemia, abnormal leukocyte (increase or decrease), abnormal platelet (increase or decrease), abnormal liver function and abnormal renal function.

#### 2.2.4. *Statistical methods*.

Application SPSS19 Statistical test for homogeneity and normality of variance, Statistics conforming to normal distribution and homogeneity of variance were expressed in *x* ± *s*, T-test was used for comparison between the 2 groups; The measurement data that did not conform to the normal distribution were expressed in the median (*P*25, *p*75),Mann–Whitney U test was used for comparison between the 2 groups; Chi square test or Fisher exact probability method were used for counting data, *a* = 0.05.

## 3. Results

### 3.1. *General information*

The total number of CwG cases included in this study was 120, including 62 males, 51.7%; and 58 females, 48.3%, and the ratio of male to female was about 1:1; The minimum age was 9 months and the maximum age was 33 months. There was no significant difference between the 2 groups in onset age, gender, course of disease, number of seizures, total number of convulsions, positive rate of rotavirus and abnormal rate of electroencephalogram (*P* > .05) (Table 1 for details).

**Table 1:** The clinical characteristics of 2 group before treatment.

Item	A group (60 cases)	B group (60 cases)	Inspection value	P value
Age (months)	17.57 ± 4.09	17.77 ± 4.21	*t = *0.08^☆^	0.78
Gender (male/ female, cases)	33/27	29/31	*χ*^*2*^ = 053^∆^	0.47
Course of disease at admission (days)	3(2, 3)	3(2, 3.5)	*Z = *0.15^▲^	0.93
Total convulsions	127	131	—	—
Once convulsions(cases)	23	21	*χ*^*2*^ = 014^∆^	0.71
Two or more convulsions(cases)	37	39		
3 days of the course of disease(cases)	49	47	*χ*^*2*^ = 0.77^∆^	0.78
more than 3 days(cases)	11	12		
rotavirus positive(cases)	16	18	*χ*^*2*^ = 0.16^∆^	0.69
abnormal EEG(cases)	13	12	*χ*^*2*^ = 0.05^∆^	0.82
Normal CT/ MRI(cases)	60	60	—	—

Note: ▲the measurement data conforming to the non normal distribution is represented by the median (P 25, P 75), and the non parametric Mann–Whitney U test is used, and the results are represented by Z value; ☆the statistics conforming to the normal distribution and homogeneity of variance are expressed in (x ± s), and the results are expressed in t value by T test; ∆chi square test was used for counting data. EEG = electroencephalogram.

### 3.2. Comparison of the effective rate

The effective rate of PB in A group was 93.33% and that in B group was 80.00%. There was significant difference between the 2 groups (*P* < .05) (Table 2 for details).

**Table 2:** Comparison of effective rates between 2 groups of PB.

Item	Cases	Effective n (%)	Ineffective n (%)
A group	60	56 (93.33)	4 (6.67)
B group	60	48 (80.00)	12 (20.00)
*χ* ^*2*^ * value*		4.62	
*P value*		0.03	

PB = phenobarbital.

All 16 children’s with recurrent convulsions after initial treatment with PB did not have convulsions after a single intravenous injection of 5 mg/kg.

### 3.3. Safety comparison

Drowsiness was the most common adverse reaction. 14 cases in A group and 7 cases in B group had drowsiness. And there was no statistical significance in the incidence of adverse events such as drowsiness, ataxia, abnormal liver function, anemia, abnormal leukocyte, respiratory depression, cognitive impairment, rash, abnormal platelet and abnormal renal function in each group (*P* > .05) (Table 3 for details).

**Table 3:** Adverse reactions or adverse events between 2 group.

Item	A group	B group	*P value*
drowsiness	14	7	0.14
ataxia	5	2	0.44
abnormal liver function	4	2	0.68
anemia	3	3	-
abnormal leukocyte	5	4	1.00
respiratory depression	0	0	-
cognitive impairment	0	0	-
rash	0	0	-
abnormal platelet	1	2	1.00
abnormal renal function	0	0	-

## 4. Discussion

In recent years, more and more article about CwG had been reported. Previous studies have shown that female are more likely to develop.^[7]^ However, this study showed that the ratio of male to female was close to 1:1. This suggested that there was no difference in incidence rate between male and female, which may be related to selective bias. The total number included in this study was 120, with the minimum age of 9 months and the maximum age of 33 months, suggesting that the disease is prone to infants and young children. Our previous studies found that 62.59% of children had 2 or more convulsions, and the incidence of convulsions in the first 3 days was as high as 84.89%.^[7]^ In this study, the proportion of cases with more than 2 seizures was 63.33%, and the incidence of convulsions in the first 3 days was as high as 80%. It suggested that we should pay more attention to whether had convulsions in the first 3 days in children with diarrhea. If have, they need active anticonvulsant treatment.

At present, there is no consensus about acute anticonvulsant drugs (drug type selection, course of treatment and dose). Although lidocaine and fosphenytoin have been reported to be effective in the treatment of the disease, in China, due to the lack of fosphenytoin, and lidocaine has not been widely accepted by the parents because it is an anesthetic. Therefore, PB is a commonly used drug for the treatment in China. Many studies, including our previous studies, have shown that PB is effective in the treatment,^[7,8,11,13–15]^ but there is still no clear conclusion on how to select the specific dose and the safety and effectiveness of different doses. In order to explore a more appropriate PB dose, this study used a prospective study to compare the efficacy of different doses of PB. It was found that the effective rate of A group was 93.33%, which was significantly higher than that of B group was 80.00% (*P* < .05), suggesting that 10 mg/kg a single dose group was better, and the children with recurrent convulsion were controlled after another single 5mg/ kg PB.

As we all know, PB has certain side effects, such as drowsiness and ataxia. Although the side effects of previous studies are temporary and reversible, the occurrence of side effects can interfere with the diagnosis of the disease and may be misdiagnosed as encephalitis, so as to further perform lumbar puncture and give corresponding treatment, which will bring unnecessary pain and burden to patients. Takami et al^[13]^ reported 24 patients, 7 of whom had narcolepsy or ataxia. This study found that 14 cases (23.3%) in A group and 7 cases (11.6%) in B group had drowsiness. Drowsiness was the most frequent adverse reaction, but there was no significant difference between the 2 groups (*P* > .05). Compared with recurrent convulsions, family members are more likely to accept the adverse reaction of drowsiness; There was no significant difference in the incidence of adverse events such as ataxia, abnormal liver function, anemia, abnormal leukocyte, respiratory depression, cognitive impairment, rash, abnormal platelet and abnormal renal function among the groups (*P* > .05). And the adverse reactions were transient. The above results further support that single dose 10mg/ kg PB can be used as the best treatment choice for CwG.

However, there are still deficiencies in this study. Our team’s previous retrospective analysis showed that about 37.5% of children had only one convulsion; There are similar reports in the study of Takami et al; Their research is to give treatment from the second seizure test, but if convulsions occur again, it will greatly increase the degree of panic and anxiety for family members. Whether to give PB treatment at the beginning of the first convulsion or the second convulsion remains to be discussed. In addition, this study only compared the efficacy and safety of CwG in the acute phase between the 2 groups, and did not compare the long-term prognosis of the 2 groups, which can be studied in future research.

In conclusion, the clinical characteristics of CwG are that it mainly occurs in infants. Two thirds of the cases can have more than 2 convulsions, and most of the convulsions occur within 3 days of the course of disease. It is recommended to apply PB 10mg/ kg once as soon as possible to prevent the recurrence of convulsions, which has high effectiveness and safety. For the cases of recurrent convulsions after injection, try to give 5mg/ kg once again.

## Acknowledgements

We gratefully acknowledge the contributions of all participants in this study, but not the list of authors. None of the authors declared any conflict of interest.

## Author contributions

**Conceptualization:** Jian Zha, Hui Chen.

**Data curation:** Yong Chen.

**Funding acquisition:** Xiongying Yu.

**Investigation:** Jihua Xie.

**Methodology:** Jian Zha, Hui Chen.

**Project administration:** Zhaoshi Yi.

**Supervision:** Jianmin Zhong.

**Writing – original draft:** Jian Zha.

**Writing – review & editing:** Hui Chen, Jianmin Zhong.

## References

[1] Lacasa MS Ramos FJ Moreno PD. Gastroenteritis-related seizures: study of incidence and clinical analysjs. An Pediatr (Barc). 2013;79:162–6. 23462096 10.1016/j.anpedi.2013.01.006

[2] Motoyama M Ichiyama T Matsushige T. Clinical characteristics of benign convulsions with rotavirus gastroenteritis. J Child Neurol. 2009;24:557–61. 19168832 10.1177/0883073808327829

[3] Kawano G Oshige K Syutou S. Benign infantile convulsions associated with mild gastroenteritis: a retrospective study of 39 cases including virological tests and efficacy of anticonvulsants. Brain Dev. 2007;29:617–22. 17544607 10.1016/j.braindev.2007.03.012

[4] Castellazzi L Principi N Agostoni C. Benign convulsions in children with mild gastroenteritis. Eur J Paediatr Neurol. 2016;20:690–5. 27292317 10.1016/j.ejpn.2016.05.014

[5] Hao XS Liang JM Wu XM. Clinical characteristics, treatment, and long-term outcomes in children suffering from benign convulsions with mild gastroenteritis: a retrospective study. BMC Pediatr. 2020;20:1–6. 33172428 10.1186/s12887-020-02406-0 PMC7656682

[6] Wu YZ Liu YH Tseng CM. Comparison of clinical characteristics between febrile and afebrile seizures associated with acute gastroenteritis in childhood. Front Pediatr. 2020;8:167. 32373562 10.3389/fped.2020.00167 PMC7176810

[7] Chen H Zhong JM Chen Y. Efficacy of phenobarbital for benign convulsions with mild gastroenteritis [in Chinese]. Jiangxi Medical Journal. 2017;52:429–32.

[8] Li T Hong S Peng X. Benign infantile convulsions associated with mild gastroenteritis: an electroclinical study of 34 patients. Seizure. 2014;23:16–9. 24125788 10.1016/j.seizure.2013.09.003

[9] Verrotti A Nanni G Agostinelli S. Benign convulsions associated with mild gastroenteritis: a multicenter clinical study. Epilepsy Res. 2011;93:107–14. 21146369 10.1016/j.eplepsyres.2010.11.004

[10] Tanabe T Okumura A Komatsu M. Clinical trial of minimal treatment for clustering seizures in cases of convulsions with mild gastroenteritis. Brain Dev. 2011;33:120–4. 20363083 10.1016/j.braindev.2010.02.007

[11] Okumura A Tanabe T Kato T. A pilot study on lidocaine tape therapy for convulsions with mild gastroenteritis. Brain Dev. 2004;26:525–9. 15533654 10.1016/j.braindev.2004.02.009

[12] Nakazawa M Toda S Abe S. Efficacy and safety of fosphenytoin for benign convulsions with mild gastroenteritis. Brain Dev.2015;37:864–7. 25708686 10.1016/j.braindev.2015.02.001

[13] Takami Y Ban H. Intravenous injection of phenobarbital for benign convulsions with mild gastroenteritis. No To Hattatsu. 2012;44:461–4. 23240527

[14] Takami Y Nakagawa T. Efficacy of phenobarbital for benign convulsions with mild gastroenteritis: a randomized, placebo-controlled trial. Brain Dev. 2019;41:600–3. 30954360 10.1016/j.braindev.2019.03.014

[15] Dura-Trave T Yoldi-Petri ME Gallinas-Victoriano F. Infantile convulsions with mild gastroenteritis: a retrospective study of 25 patients. Eur J Neurol. 2011;18:273–8. 20618844 10.1111/j.1468-1331.2010.03120.x

